# Perineal burn contractures: An experience in tertiary hospital of a Himalayan State

**DOI:** 10.4103/0970-0358.39666

**Published:** 2008

**Authors:** Jagdeep S. Thakur, C. G. S. Chauhan, Vijay K. Diwana, Dayal Chand Chauhan, Anamika Thakur

**Affiliations:** 1 Departments of Plastic and Reconstructive Surgery, IG Medical College, Shimla-171 001, HP, India; Department of Pharmacology, IG Medical College, Shimla-171 001, HP, India

**Keywords:** Burn, contracture, perineum

## Abstract

Perineal burn contracture is a rare burn sequel. We conducted a retrospective analysis of cases with perineal burn contractures managed in a tertiary care centre of a Himalayan state. We found that all cases sustained burn injury from burning firewood and the time of presentation was two to six years after the burn injury. We analyzed our treatment method and have classified these contractures into two types.

## INTRODUCTION

Every year there are about 0.7–0.8 million hospital admissions with burns in India[1] as compared to about 13,000 in the UK.[2] This major difference shows the gravity of this problem in India where there are very few hospitals with separate burn units. Hence, most of the burn patients are being managed by staff without special training in burn care. The management of acute burns is a challenge to everyone from burn specialists to paramedics. The aim of the management of a burn patient is to provide a life without any disability and this requires a lot of hard work by the hospital personnel as well as cooperation from both the patients and their family members.

The perineum is a very important site in the body anatomically and functionally. Fortunately though, burn contractures here are rare. The perineum usually escapes burn injury due to its deep location between the thighs. Most often, it is the burn contracture of the surrounding area, *i.e.*, the lower part of the abdomen, the inguinal area, and the adjacent thighs that secondarily distorts the perineum, less often it is the perineum that is primarily burnt and can, if not treated appropriately, result in contracture.

We conducted a retrospective analysis of patients with perineal burn contractures and present our experience and a method of treating these cases in a tertiary care center in Himachal Pradesh

## MATERIALS AND METHODS

**Figure 1:**
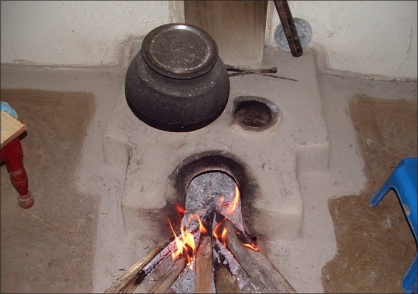
A typical ‘Chullah’ found in HP

We reviewed the records of patients with burn sequalae managed in our department from January 2003 to December 2007. There were 237 patients with various burn squeal in this five year period. Out of these 237 patients, there were 182 patients who had sustained flame burns in childhood and had burn sequalae in different parts of the body, mainly in the upper limbs (96 cases). However, perineal burn contractures were found only in six patients [Table 1]: five females and one male, all aged 6–15 years. Difficulty in squatting for the last two to nine years was the main complaint in all cases. All six patients suffered burns from burning firewood used in the “Chullah” [Figure 1].

**Table 1:** Details of patients with etiology and duration of burns

*Case No.*	*Age (Years)*	*Sex*	*Mode of injury*	*Percentage of burn*	*Duration of burn (Years)*
1	6	Female	Accidentally sat on the hot chullah	20	4 years
2	15	Female	Fall of spark on the clothes while cooking	25	4.5 years
3	14	Male	Same as case no 2	10	2 years
4	8	Female	Fell on burning wood	25	5 years
5	10	Female	Fall of spark on the clothes in the kitchen	24	9 years
6	12	Female	Same as case no 5	18	7 years

We have classified these contractures into primary and secondary perineal burn contractures:

*Primary perineal burn contractures* are rare. In these, the perineum actually sustains burns. The ensuing contracture pulls the external genitals and anus towards each other. In addition, the scar binds the two thighs together.

*Secondary perineal burn contractures* are more common. In this case, the perineum is affected because of the pull of the scars in the surrounding areas. The external genital organs are usually hidden from view.

**Figure 2:**
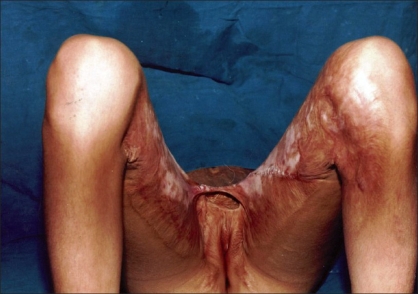
Perineal contracture leading to inability to abduct thighs

**Figure 3:**
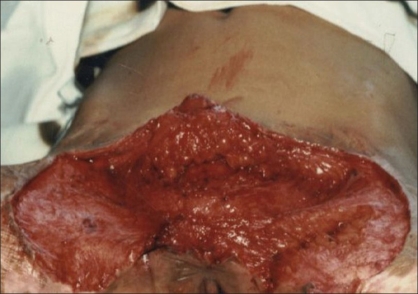
Raw area after excision of contracture

**Figure 4:**
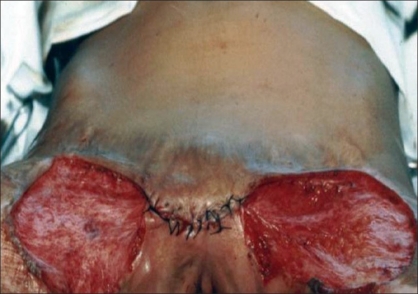
Closer with advancement of skin of abdomen

In our patients, five females had secondary contractures and the single male had a primary contracture as defined above. All these patients underwent surgery under general anesthesia and the surgical technique used was as follows:

In five patients, contractures [Figure 2] were released widely which created a large raw area [Figure 3]. Wide undermining of the abdominal skin flap was subsequently done to facilitate its advancement downwards to cover the raw area in front of the pubis [Figure 4]. The raw area on either side was covered with sheets of a thick split-thickness skin graft and tie-over dressings were applied to immobilize the graft.

**Figure 5:**
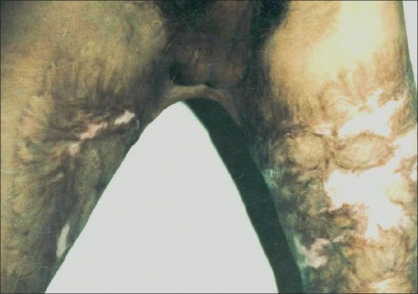
Male patient with contracture

The sixth (male) patient [Figure 5] did not require a skin graft and the distortion of the external genitalia was corrected and restored to its normal position. The resulting raw area was covered by mobilizing the scrotal skin and the adjacent thigh skin.

**Figure 6:**
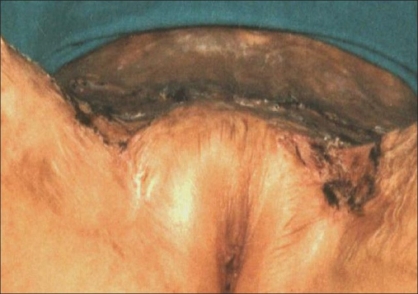
Well healed surgical site

**Figure 7:**
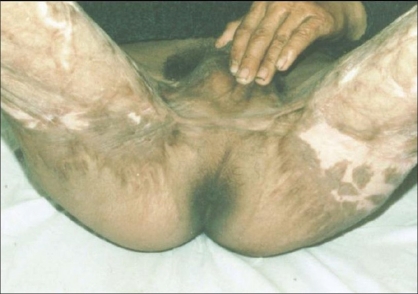
Postoperative photograph of male patient

Postoperatively, indwelling urethral catheter drainage was instituted for three days and the area healed well with total take up of the graft [Figures 6 and 7]. Postoperatively, the patients were asked to wear tightly fitting undergarments with sponge padding to prevent contraction of the grafted area. The follow-up period ranged from six months to four years

## RESULTS

All patients healed well and were functionally restored. There were minor recurrences in terms of secondary contraction of graft, in two patients but these were managed by physiotherapy. Squatting ability improved in all and they were able to perform essential chores that require squatting position.

## DISCUSSION

Commonly, patients seek consultation for reconstruction of post burn contractures of the hand, neck, and lower limb. Minor perineal burn contractures which can be unsightly are normally ignored by the patients as they are well hidden by clothes. However, these perineal burn contractures can cause a functional disability. The thick scar bands across the symphysis pubis behind the genitals can bind the thighs together, leading to impairment of the movement of the hip joints, especially abduction. Due to this limitation in movement, walking, sitting, urination, defecation, and sexual intercourse become difficult. Squatting, a common posture adopted in India for urination and defecation, becomes extremely difficult and in fact, this was the main complaint in all our cases.

In females, genital organs do not sustain deep burns due to their peculiar location, while males escape these contractures due to the normal laxity and redundancy of penile and scrotal skin. In essence this allows for excess skin availability to compensate for loss.

The mode of injury in all our cases was different from reported modes of injury, *i.e.*, kerosene burns and hot water burns.[1–11] Sawhney[3] had reported six children with perineal burns that had been sustained by spilling of kerosene on the clothes from a burning stove or due to the explosion of such stoves. In 1994, Gupta *et al.*[5] reported an epidemic in Rajasthan of burns caused by using kerosene mixed with petrol in pressure stoves. Weiler-Mithoff *et al.*[6] reported an unusual case of burn injury to the genital area by fireworks in a suicide attempt. Michielsen *et al.*[7] reported burn injury by hot fluid (55%), flame (24%), and chemicals (16%) in a study of 4,216 patients, while scalds were responsible for 85% burns in a review of 27 cases by the same authors.[8] **Quayle** *et al.*[9] found increased burn injury rates in African-American girls (0–4 years of age) in counties with a high poverty rate in Missouri (USA). In a retrospective ten year study of 309 children, Kumar *et al.*[10] reported scalds (72.5%), flames (22.7%), and electric burns (3.2%) as the most_common contributor to burn injuries. In another study done in India on pediatric burn cases,[11] scalds were found to be common in children younger than six years of age while flame and electric burns were predominant in children aged 6–14 years.

The difference in the mode of injury in our patients was due to the common use of wood as fuel for cooking and heating in Himachal Pradesh. This state is located in the Himalayan ranges with 95% of the population living in villages surrounded by thick forest. The majority of these villages are located in far flung snow-bound areas with poor road connectivity, leading to unavailability of Liquid Petroleum Gas (LPG) cylinders. Although kerosene pressure stoves are frequently used by villagers or economically poor families for cooking in India, the use of the chullah is a common practice seen in this state. Due to poor economical status, cold climate, and easy availability of wood, the chullah is widely used for cooking and heating. People are in the habit of sitting in front of burning wood placed in the chullah for warmth and this can lead to fall of sparks on the clothes and burn injury primarily in the lower body due to its closeness to the fire.

As stated by Ahuja and Bhattacharya,[1] the use of loose clothes during cooking is the major cause for burn injury but as the cases in our study were children, negligence on the part of family members appeared to be the cause. This observation is contradictory to the findings of Ahuja and Bhattacharya,[1] who reported that children are less prone to burn injury as they are looked after by adults in joint families.

Extensive raw areas are produced on release of the contractures. While resurfacing, it is desirable to provide a full thickness skin cover over the symphysis pubis as it breaks the continuity of the skin grafted area. However, this may not be possible when the abdominal skin is deeply scarred. Long-term measures have to be instituted postoperatively to prevent skin graft contraction such as wearing tightly fitting undergarments as reported by Sawhney[3] and Rutan.[12] This long-term measure is the main drawback of this technique but one has to contend with all the problems associated with split thickness skin grafts in the absence of alternatives.

We have treated all five cases of secondary perineal burn contractures with the technique mentioned earlier, with consistently good results. The lone patient with primary perineal burn contracture as mentioned earlier, did not require any skin graft.

As reported by other authors,[1,13–16] the management of burn patients in the developing world is different from that in the developed world due to lack of education, funds, burns units, and untrained staff. Hence, improvement has to make in these areas, especially in encouraging people to use LPG stoves, as their use not only reduces the chances of these types of accidents but also conserves the environment, a major issue in this decade. Educating people for early medical consultation and proper postoperative care and rehabilitation can prevent these burn sequalae.
